# Early Transplant Outcomes and Kidney Donor Profile Index Risk Prediction Performance in Kidneys Recovered Using a Uniform Abdominal Normothermic Regional Perfusion Technique

**DOI:** 10.1111/ctr.70729

**Published:** 2026-10-23

**Authors:** Motaz A. Selim, Michelle Buff, Michael Fenlon, Austin Forbes, Chinedu Nwaduru, Leonardo Aviles Ovalle, Rebecca Kohler, Joshua J. Horns, Jean Botha, Michael A. Zimmerman

**Affiliations:** ^1^ Division of Transplant Surgery, Department of Surgery University of Utah Salt Lake City Utah USA; ^2^ Department of Surgery University of Utah Salt Lake City Utah USA; ^3^ Division of Transplant Surgery, Department of Surgery Intermountain Health Care Salt Lake City Utah USA

**Keywords:** abdominal normothermic regional perfusion, donation after circulatory death, delayed graft function, kidney transplantation, KDPI, kidney nonuse, organ utilization

## Abstract

**Background:**

Abdominal normothermic regional perfusion (aNRP) may mitigate ischemic injury in donation after circulatory death (DCD). Specific aNRP‐DCD kidney transplant (KTx) outcomes are inadequately defined. Kidney Donor Profile Index (KDPI) assigns equivalent DCD risk components to all DCD kidneys and may not capture procurement‐related differences.

**Methods:**

We retrospectively analyzed KTx from donors in a single donation service area procured between January 2023‐December 2024. DCD‐restricted multivariable logistic and Cox models compared aNRP‐DCD with super‐rapid recovery (SRR)‐DCD and evaluated KDPI associations with delayed graft function (DGF) and 1‐year overall graft survival.

**Results:**

523 KTx (310 from brain‐dead donors (BDD), 122 from SRR‐DCD, 91 from aNRP‐DCD). aNRP‐DCD kidneys came from older, higher‐KDPI donors than SRR‐DCD and BDD (Age 55 vs 43.5 vs 38 years; *p* = 0.01, *p* < 0.001; KDPI 66% vs 53% vs 27%; *p* = 0.008, *p* < 0.001). In DCD‐restricted multivariable analysis, aNRP was associated with lower DGF odds than SRR (OR 0.445, 95% CI 0.23‐0.861; *p* = 0.016). KDPI was associated with DGF (OR 1.028, 95% CI 1.014‐1.042; *p* < 0.001). One‐year overall graft survival was similar. KDPI interaction modeling suggested lower DGF odds at comparable KDPI values, however the KDPI × donor‐type interaction was not statistically significant.

**Conclusions:**

In this uniform cohort, aNRP had lower DGF odds than SRR despite higher risk. KDPI‐DGF interaction patterns were concordant in SRR‐DCD kidneys but less clear in aNRP‐DCD; however, formal interaction was not significant. These findings suggest that aNRP‐DCD may modify early graft risk; however, larger studies with longer follow‐up are needed to determine whether aNRP‐DCD affects longer‐term outcomes or KDPI performance.

**Abbreviations**
aNRP‐DCD: abdominal normothermic regional perfusion donation after circulatory death
AKI: acute kidney injury
BDD: brain‐dead donor
BMI: body mass index
CI: confidence interval
CIT: cold ischemia time
CKD‐EPI: Chronic Kidney Disease Epidemiology Collaboration
DCD: donation after circulatory death
DGF: delayed graft function
DSA: donation service area
dWIT: donor warm ischemic time
ECMO: extracorporeal membrane oxygenation
eGFR: estimated glomerular filtration rate
fWIT: functional warm ischemic time
HR: hazard ratio
HRSA: Health Resources and Services Administration
IQR: interquartile range
IRB: institutional review board
KDPI: Kidney Donor Profile Index
KTx: kidney transplant
NMP: normothermic machine perfusion
NRP: normothermic regional perfusion
OPO: organ procurement organization
OPTN: Organ Procurement and Transplantation Network
OR: odds ratio
SRR‐DCD: super‐rapid recovery donation after circulatory death
STAR: Standard Transplant Analysis and Research
STROBE: Strengthening the Reporting of Observational Studies in Epidemiology
TANRP: thoracoabdominal normothermic regional perfusion
WOLS: withdrawal of life‐sustaining support

## 1 Introduction

The persistent shortage of transplantable kidneys remains a major driver of waitlist dropout in the United States; more than 8,000 patients were removed from the kidney waitlist in 2023 because of death or becoming too sick for transplantation, while kidney nonuse remains substantial (27.9%), particularly among kidneys with high Kidney Donor Profile Index (KDPI) (72.5%) and donation after circulatory death (DCD) kidneys (34.8%) [1]. Because DCD donors now comprise a growing share of the deceased donor pool, nearing 50% [2], improving the performance and utilization of DCD kidneys has become increasingly important.

Conventional super‐rapid recovery DCD (SRR‐DCD) procurements incur donor warm ischemic injury and are associated with almost twice the rates of delayed graft function (DGF) compared to brain‐dead donor (BDD) kidneys [3, 4]. DGF not only imposes logistic and financial burdens [5], but also affects long‐term graft survival [6, 7].

Normothermic regional perfusion (NRP) emerged as a strategy that may mitigate the DCD warm ischemic injury by restoring oxygenated circulation before cold preservation [8]. Early studies suggest improved organ yield, reduced DGF and better utilization compared with conventional SRR‐DCD procurements [9, 10]. Although abdominal NRP (aNRP) has expanded rapidly in contemporary US noncardiac DCD procurements [11], the US published kidney outcomes literature has only recently begun to address this shift [10, 12]. Most earlier US outcome reports largely reflect thoraco‐abdominal NRP (TANRP) or mixed NRP cohorts [9, 13, 14], with much of the more specific aNRP experience still coming from European experience [15, 16].

Overall, interpretation of the current kidney transplant NRP literature remains limited by heterogeneity in procurement techniques. Many reports either focus only on TANRP or combine aNRP and TANRP as one cohort despite their physiological and donor‐selection differences. TANRP donors are often selected within potential heart donation and therefore tend to be younger with lower KDPI than aNRP donors [13, 14]. The two techniques may also exert a different physiologic effect on organs because TANRP restores pulsatile, cardiac‐driven circulation, whereas aNRP relies on pump‐driven, nonpulsatile reperfusion [17]. Thus, analyses that pool these groups may obscure the specific performance profile of aNRP kidneys. Published registry‐based analyses rely on indirect inference to identify NRP donors because national datasets did not explicitly distinguish NRP from SRR‐DCD procurement until late 2025. Even with that, national data still does not reliably distinguish aNRP from TANRP, capture granular run‐level perfusion parameters, or account for unsuccessful NRP attempts converted to standard recovery. These limitations may introduce misclassification, selection, and information bias, weakening the validity and interpretability of aNRP‐specific comparisons [9, 12, 18].

The increasing use of NRP raises another important question about the interpretation of Kidney Donor Profile Index (KDPI) in this setting. KDPI is described by the Organ Procurement and Transplantation Network (OPTN) as a calculation used to “summarize into a single number the quality of deceased donor kidneys relative to other recovered kidneys” [19]. Recent work has highlighted two distinct but related concerns with this framework. First, KDPI labeling effect has long been debated as a potential contributor to kidney nonuse, particularly in higher‐KDPI organs [20, 21]. Second, because KDPI is a percentile‐based measure that is rescaled yearly to a shifting donor pool, the same KDPI value may not represent the same underlying donor risk over time, raising concerns about calibration drift and “moving goalposts” in clinical interpretation [22, 23, 24]. This may be especially relevant in DCD kidneys, where the current KDPI formula applies the same DCD risk component to aNRP and conventional SRR‐DCD kidneys [19] despite their distinct procurement physiology and potentially different early graft behavior after transplantation.

Accordingly, we evaluated early transplant outcomes in kidneys recovered using a uniform aNRP technique and examined the relationship between KDPI and early outcomes including DGF and one‐year overall graft survival. A secondary aim was to assess whether KDPI appeared to reflect early post‐transplant risk similarly across aNRP‐DCD and SRR‐DCD kidneys.

## 2 Methods

### 2.1 Study Design and Data Sources

This retrospective cohort study evaluated kidney transplants from a single donation service area (DSA) recovered between January 2023 and December 2024. Identified donor data was requested from OPTN for all donors from a single donation service area during the study period and cross‐referenced with internal procurement records to identify aNRP donors and to collect granular aNRP run characteristics.

The analytic transplant cohort was restricted to single kidney transplants from BDD, SRR‐DCD, and completed aNRP‐DCD. We excluded TANRP donors, aborted or incomplete aNRP runs, en‐bloc kidney transplants, and dual kidney transplants.

Recipient, donor, and transplant‐level variables were obtained from the OPTN Standard Transplant Analysis and Research (STAR) files. Outcomes including cold ischemia time (CIT), DGF, graft survival, and patient survival were derived from STAR recipient outcome fields. (Figure S1)

### 2.2 ANRP Technique

Local aNRP runs during this period used uniform techniques and parameters. Donor management and DCD protocols, including withdrawal of life‐sustaining support (WOLS) and independent death declaration, were conducted in accordance with established institutional and national standards [25, 26]. Systemic anticoagulation was administered prior to WOLS in accordance with institutional protocols. Death was declared by an independent physician after cessation of circulatory functions followed by the mandated 5‐minute observation period. The uniform aNRP protocol included an abdominal incision with abdominal aorta and inferior vena cava cannulation, cross‐clamping of the supra‐celiac or descending thoracic aorta via sternotomy, and initiation of normothermic oxygenated blood flow using a centrifugal pump targeting flows >2 L/min/m^2^. Oxygenation was provided via a membrane oxygenator incorporated into the extracorporeal circuit. Circuit temperature was maintained using an external heat exchanger, with a target perfusate temperature of 36–37°C to achieve normothermia. Packed red blood cells were transfused to maintain a hemoglobin goal >7 g/dL. Standard run time was 120 min with serial lactate and pH monitoring at baseline and every 30 min thereafter. Successful NRP runs were assessed primarily by visualization of adequate bowel and liver perfusion and maintenance of target flow for >90% of the run time. Donor warm ischemic time (dWIT), defined as the interval from withdrawal of life support to declaration of death, was capped at 120 min per local protocols. Functional warm ischemic time (fWIT) was defined as the interval from donor systolic blood pressure below 80 mmHg to initiation of NRP. We also added a calculated fWIT based on systolic blood pressure below 50 mmHg for the sake of comparison.

### 2.3 ANRP Run Data

Perfusion parameters were recorded for aNRP runs performed in donors intended for kidney procurement, regardless of whether the kidneys were ultimately transplanted. These included perfusion flow, duration, urine output, and metabolic parameters (lactate and pH) as well as kidney and liver utilization. Donor and perfusion characteristics were evaluated between completed aNRP runs that yielded at least one transplanted kidney and those in which both kidneys were not utilized. Donor characteristics were analyzed at the donor level, with each donor contributing a single observation regardless of the number of kidneys transplanted.

### 2.4 Organ Utilization Analysis

Kidney nonuse was calculated across the study groups using all potential kidneys from BDD, SRR‐DCD, and completed aNRP‐DCD donors from whom kidneys were recovered with the intent of transplantation.

### 2.5 Kidney Transplant Outcomes

The primary outcomes were DGF, defined as dialysis within 7 days of transplant per OPTN criteria, and overall graft survival at one year. The secondary outcome was the association between KDPI and DGF.

### 2.6 Analytic Strategy

We performed two complementary analytic approaches:

First, we performed full transplant‐cohort univariate and survival analyses including BDD, SRR‐DCD, and aNRP‐DCD recipients. Baseline characteristics were compared. Overall graft survival was estimated using Kaplan–Meier methods and compared with the log‐rank test.

Second, we performed DCD‐restricted multivariable analyses limited to SRR‐DCD and aNRP‐DCD recipients. Multivariable logistic regression was used to evaluate DGF, and Cox proportional hazards modeling was used to evaluate the overall graft survival within one year of transplant.

This approach was designed to directly compare the two DCD procurement modalities while evaluating the relationship between KDPI and outcomes. Excluding BDD kidneys avoided confounding and double adjustment from differential weighting of BDD versus DCD donor type within KDPI, allowing a clearer assessment of KDPI–outcome relationships and interaction with aNRP versus SRR procurement modality which share the same DCD weight in KDPI calculation.

### 2.7 KDPI Performance Analysis

Multivariable model‐predicted probabilities for DGF and one‐year overall graft survival were compared with observed event rates across the KDPI range. Interaction models including KDPI × donor type were constructed to assess whether the relationship between KDPI and outcomes differed by procurement strategy within the DCD‐restricted cohort.

### 2.8 Statistical Analysis

Continuous variables are presented as median (interquartile range) and categorical variables as counts (percentages). Group comparisons were performed using Mann–Whitney U or Kruskal–Wallis tests for continuous variables and chi‐squared or Fisher's exact tests for categorical variables. Estimated glomerular filtration rate (eGFR) at 6 and 12 months was calculated among recipients aged ≥18 years using the race‐free 2021 Chronic Kidney Disease Epidemiology Collaboration (CKD‐EPI) creatinine equation incorporating serum creatinine, age, and sex.

Multivariable logistic regression models were constructed to evaluate factors associated with DGF. Covariates included donor, recipient, and transplant characteristics. Models were refined using elastic net regression to identify the most informative variables.

Graft survival was analyzed using time from transplant to graft loss or patient death. Kaplan–Meier methods were used to estimate event‐free survival, with comparisons across donor types performed using the log‐rank test. Cumulative incidences of death and death‐censored graft loss were estimated at 30, 180, and 365 days after transplant by procurement type using competing‐risks analysis, with the alternate event treated as a competing risk and 95% confidence intervals calculated for each time point.

A multivariable Cox proportional hazards model was constructed to evaluate the association between donor type and the graft survival outcome, adjusting for the same covariates retained in the final DGF model. Patients were censored at last follow‐up or at one‐year post‐transplant, whichever occurred first.

Statistical analyses were performed using R (version 4.4.0; R Core Team, Vienna, Austria) [27].

This study used data from the Organ Procurement and Transplantation Network (OPTN). The OPTN data system includes data on all donor, wait‐listed candidates, and transplant recipients in the US, submitted by the members of the Organ Procurement and Transplantation Network (OPTN). The Health Resources and Services Administration (HRSA), U.S. Department of Health and Human Services provides oversight to the activities of the OPTN contractor.

### 2.9 Ethics and Reporting

The study was approved by the Institutional Review Board (IRB) of the University of Utah (IRB #00185857) and conducted in accordance with the Declaration of Helsinki. The requirement for informed consent was waived because of the retrospective design and use of existing clinical and registry data. Study findings were reported in accordance with the Strengthening the Reporting of Observational Studies in Epidemiology (STROBE) guideline [28].

## 3 Results

Between January 2023 and December 2024, 400 donor procurements were performed locally yielding 566 kidney transplants. 523 kidney transplants met inclusion criteria and were analyzed: 310 from BDD, 122 from SRR‐DCD, and 91 from aNRP‐DCD. (Figure S1) Median follow‐up was 350 days (IQR 169‐396). During follow‐up, a total of 122 cases of DGF (23.3%), 20 graft losses (3.8%), and 10 deaths (1.9%) were observed.

### 3.1 Baseline Demographics and Clinical Variables

Median donor age was significantly higher in aNRP‐DCD (55 years, IQR 33‐62) compared with SRR‐DCD (43.5, IQR 29‐57, *p* = 0.01) and BDD (38, IQR 27‐52, *p* < 0.001). Median donor KDPI was also significantly higher for aNRP‐DCD: 66 (IQR 38‐85) versus SRR‐DCD (53, IQR 33‐76, *p* = 0.008) and versus BDD (27, IQR 10‐50, *p* < 0.001). Other donor and recipient characteristics are summarized in Table 1.

**TABLE 1:** Baseline Donor and Recipient Characteristics by Procurement Type.

	aNRP‐DCD (*n* = 91)	SRR‐DCD (*n* = 122)	BDD (*n* = 310)	aNRP vs SRR (p)	aNRP vs BDD (p)	SRR vs BDD (p)
Donor age, years. Median (IQR)	55 (33–62)	43.5 (29–57)	38 (27–52)	0.012	<0.001	0.054
Donor gender, male. N (%)	54 (59.34)	67 (54.9)	186 (60)	0.6	1	0.39
Donor BMI, kg/m^2^. Median (IQR)	28.77 (22.32–33.19)	29.3 (24.71–35.81)	27.96 (23.91–31.2)	0.38	0.93	0.008
Donor AKI. N (%)	12 (13.2)	16 (13.1)	54 (17.4)	1	0.4	0.34
Donor KDPI, %. Median (IQR)	66 (38–85)	53 (33–76)	27 (10–50)	0.008	<0.001	<0.001
Recipient age, years. Median (IQR)	58 (39–65)	58 (44.3–66)	52.5 (37–63)	0.54	0.08	0.005
Recipient gender, male. N (%)	56 (61.5)	81 (66.4)	178 (57.4)	0.55	0.56	0.1
Recipient BMI, kg/m^2^. Median (IQR)	30.46 (24.93–34.61)	28.05 (23.88–31.8)	28.76 (23.62–33.44)	0.025	0.09	0.38
Cold ischemia time, hours. Median (IQR)	18.1 (14.4–22.7)	21.0 (17.1–24.8)	19.2 (14.2–23)	0.01	0.97	0.002
Pumped. N (%)	91 (100)	122 (100)	287 (92.6)	1	0.02	0.004
Length of stay, days. Median (IQR)	3 (3–5)	3.5 (3–5)	4 (3–5)	0.96	0.2	0.2
Delayed graft function. N (%)	26 (28.57%)	47 (38.52%)	49 (15.81%)	0.17	0.01	<0.001
6‐month eGFR, mL/min/1.73 m^2^, median (IQR)	52.6 (41.4–72.9)	54.3 (43.1–68.1)	62.3 (48.2–76.4)	0.78	0.02	0.002
Available for analysis, n (%)	89 (97.8)	116 (95.1)	292 (94.2)			
12‐month eGFR, mL/min/1.73 m^2^, median (IQR)	52.8 (43.3–78.3)	59.4 (41.4–68.6)	62.9 (48.6–82.3)	0.85	0.051	0.004
Available for analysis, n (%)	58 (63.7)	93 (76.2)	233 (75.2)			

Abbreviation: AKI: acute kidney injury, aNRP‐DCD: abdominal normothermic regional perfusion‐donation after circulatory death, BDD: brain‐dead donor, BMI: body mass index, eGFR: estimated glomerular filtration rate, KDPI: Kidney Donor Profile Index, SRR‐DCD: super‐rapid recovery‐donation after circulatory death.

### 3.2 Kidney Utilization and Nonuse

Across the three groups, 352 donors in whom kidneys were procured with the intent of transplant represented 704 potential kidneys. Overall, 523 kidneys were transplanted and 181 were not used, corresponding to an overall kidney nonuse rate of 25.7%.

By group, BDD accounted for 376 potential kidneys, with 310 transplanted and 66 nonused (17.6%). SRR‐DCD accounted for 186 potential kidneys, with 122 transplanted and 64 nonused (34.4%). Among 74 attempted aNRP procurements, 2 incomplete and 1 aborted run were excluded. The remaining 71 completed aNRP‐DCD donors accounted for 142 potential kidneys, of which 91 were transplanted and 51 were nonused (35.9%).

Kidney nonuse was significantly lower in BDD compared with both SRR‐DCD and completed aNRP‐DCD donors (*p* < 0.001 each), and did not differ significantly between SRR‐DCD and completed aNRP‐DCD donors (*p* = 0.816). (Table S1)

### 3.3 ANRP‐DCD Run Outcomes

Of 74 attempted aNRP donors, 3 were not completed. One case was aborted upon identifying metastatic adenocarcinoma during procurement. The other two donors were converted to SRR due to poor flows with inadequate bowel perfusion on visual assessment. Among these two donors converted to SRR, both kidneys were ultimately not utilized.

Of the remaining 71 completed aNRP runs, 51 donors yielded at least one kidney transplant, resulting in 91 kidney transplants, and 20 donors had bilateral kidney nonuse.

### 3.4 Donor and aNRP Run Characteristics Associated with Kidney Utilization in aNRP Donors

Compared to aNRP donors with at least one kidney utilized (*n* = 51), aNRP donors with bilateral kidney nonuse (*n* = 20) were older (65 [62–69] vs 55 [36–62] years, *p* < 0.001), with higher KDPI (94% [88–98] vs 67% [40–85], *p* < 0.001), and a higher incidence of diabetes and AKI. aNRP run characteristics were largely similar between the two groups, including functional warm ischemia time, perfusion duration, lactates, and urine output during perfusion. Average aNRP flow was modestly lower in the bilateral nonuse group (2.40 [2.28–2.80] vs 2.75 [2.50–3.20] L/min/m^2^, *p* = 0.015), yet visual assessment of abdominal organs was considered acceptable in both groups. Terminal pH was also lower in the bilateral nonuse group (7.23 [7.15–7.30] vs 7.37 [7.25–7.44], *p* = 0.022). (Table 2)

**TABLE 2:** Donor and aNRP Run Parameters in Completed aNRP Runs with Kidney Utilization Versus Bilateral Kidney Nonuse. Donor characteristics were analyzed at the donor level, with each donor contributing a single observation regardless of the number of kidneys transplanted. Between‐group comparisons were performed using the Mann–Whitney U test for continuous variables and Fisher's exact test for categorical variables.

	Bilateral Kidney Nonuse *N* = 20	Kidney Used *N* = 51	*p* value
KDPI (%)	94 (88‐98)	67 (40‐85)	<0.001
Donor age, year. Median (IQR)	65 (62‐69)	55 (36‐62)	<0.001
Donor diabetes, yes. N (%)	7 (35)	7 (13)	0.054
Donor AKI. N (%)	9 (45)	7 (13.7)	0.009
Donor BMI. Median (IQR)	28.8 (23.9‐33.9)	28.9 (22.5‐33.3)	0.59
fWIT80, minutes. Median (IQR)	27 (26‐38)	28 (24‐37)	0.81
fWIT50, minutes. Median (IQR)	26 (23‐31)	24 (21‐32)	0.4
aNRP flow time, minutes. Median (IQR)	122 (112‐132)	120 (112‐127)	0.76
Flow, L/min/m2. Median (IQR)	2.40 (2.28‐2.80)	2.75 (2.50, 3.20)	0.015
Initial Lactate, mg/dL. Median (IQR)	12.8 (11.3‐14.5)	13.7 (11.3‐16.1)	0.36
Terminal Lactate, mg/dL. Median (IQR)	5.6 (3.6‐11.8)	6.5 (4.2‐8.2)	0.76
Initial pH. Median (IQR)	7.00 (6.80‐7.04)	7.00 (7.00‐7.11)	0.062
Terminal pH. Median (IQR)	7.23 (7.15‐7.3)	7.37 (7.25‐7.44)	0.022
Urine output, mL. Median (IQR)	0 (0‐125)	0 (0‐44)	0.75

Abbreviation: aNRP, abdominal normothermic regional perfusion; AKI, acute kidney injury; BMI, body mass index; fWIT50, functional warm ischemic time from systolic blood pressure <50 to start of NRP flow; fWIT80, functional warm ischemic time systolic blood pressure <80 to start of NRP flow; IQR, interquartile range; KDPI, Kidney Donor Profile Index.

KDPI‐adjusted analyses did not identify independent associations between individual aNRP run parameters and DGF or bilateral kidney nonuse. (Table S3)

### 3.5 Outcomes

#### 3.5.1 DGF

In DCD‐restricted multivariable analysis, aNRP was independently associated with lower odds of DGF compared with SRR (OR 0.445, 95% CI 0.23–0.861, *p* = 0.016). Higher KDPI (OR 1.028 per point, 95% CI 1.014–1.042, *p* < 0.001) was independently associated with DGF.

#### 3.5.2 Overall Graft Survival

For overall graft survival at one year, there was no statistically significant difference between aNRP and SRR across the KDPI range (HR 0.462, 95% CI 0.077–2.757; *p* = 0.397). KDPI was likewise not significantly associated with the overall graft survival outcome in the DCD‐restricted model.

Kaplan–Meier analysis of graft survival demonstrated no significant difference among BDD, aNRP‐DCD, and SRR‐DCD recipients (log‐rank *p* = 0.86). Pairwise comparisons were similarly non‐significant (aNRP vs BDD *p* = 0.75; aNRP vs SRR *p* = 0.71; BDD vs SRR *p* = 0.72). One‐year event‐free survival was 97% for BDD, 97% for aNRP‐DCD, and 96% for SRR‐DCD recipients. (Figure 1A)

**FIGURE 1:**
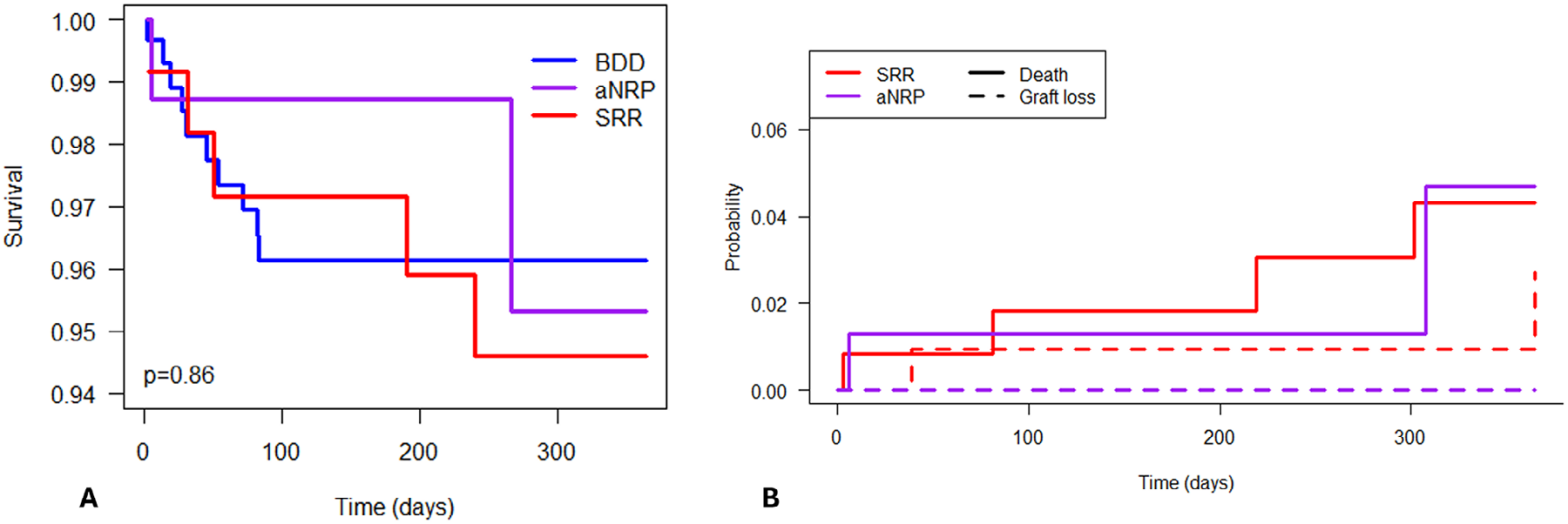
One‐year patient and graft outcomes (A). Kaplan–Meier curve for overall graft survival across the three groups. (B). Competing‐risk curves showing death and graft loss as separate events among DCD kidney recipients stratified by procurement strategy. aNRP, abdominal normothermic regional perfusion; BDD, brain dead donor; DCD, donation after circulatory death; SRR, super‐rapid recovery.

Using competing‐risks analysis, the cumulative incidence of death was low and similar between DCD groups at 30, 180, and 365 days, whereas death‐censored graft loss remained uncommon overall but was higher in the SRR group by 180 and 365 days. (Figure 1B)

#### 3.5.3 EGFR

Median eGFR was similar between aNRP‐DCD and SRR‐DCD at 6 and 12 months. BDD recipients had higher eGFR overall, although the aNRP versus BDD difference at 12 months did not reach statistical significance (*p* = 0.051). (Table 1)

#### 3.5.4 Rejection

Rejection incidence within 6 and 12 months did not differ between groups. Among recipients with available data, treated rejection occurred in 2/74 (2.7%) aNRP, 4/101 (4.0%) SRR‐DCD, and 13/269 (4.8%) BDD at 6 months, and in 3/53 (5.7%) aNRP, 5/88 (5.7%) SRR‐DCD, and 19/218 (8.7%) BDD at 12 months. (Table S2)

#### 3.5.5 KDPI Risk Prediction Concordance Models

##### 3.5.5.1 KDPI Concordance With DGF in the DCD‐Restricted Model

In the multivariable interaction model for DGF, KDPI was associated with higher odds of DGF (OR 1.019 per 1% increase, 95% CI 1.002–1.036; *p* = 0.025). The predicted probability curves showed that aNRP‐DCD kidneys appeared to have lower DGF risk than SRR‐DCD kidneys at a given KDPI level across most of the observed range. However, the formal KDPI × donor‐type interaction did not reach statistical significance (OR 1.025, 95% CI 0.996–1.056; *p* = 0.094). (Figure 2A) We further explored alternative KDPI sensitivity models in the DCD‐restricted cohort, including continuous 1‐point and 20‐point KDPI increments and a KDPI ≥85% threshold model; these showed broadly similar results. (Table S4)

**FIGURE 2:**
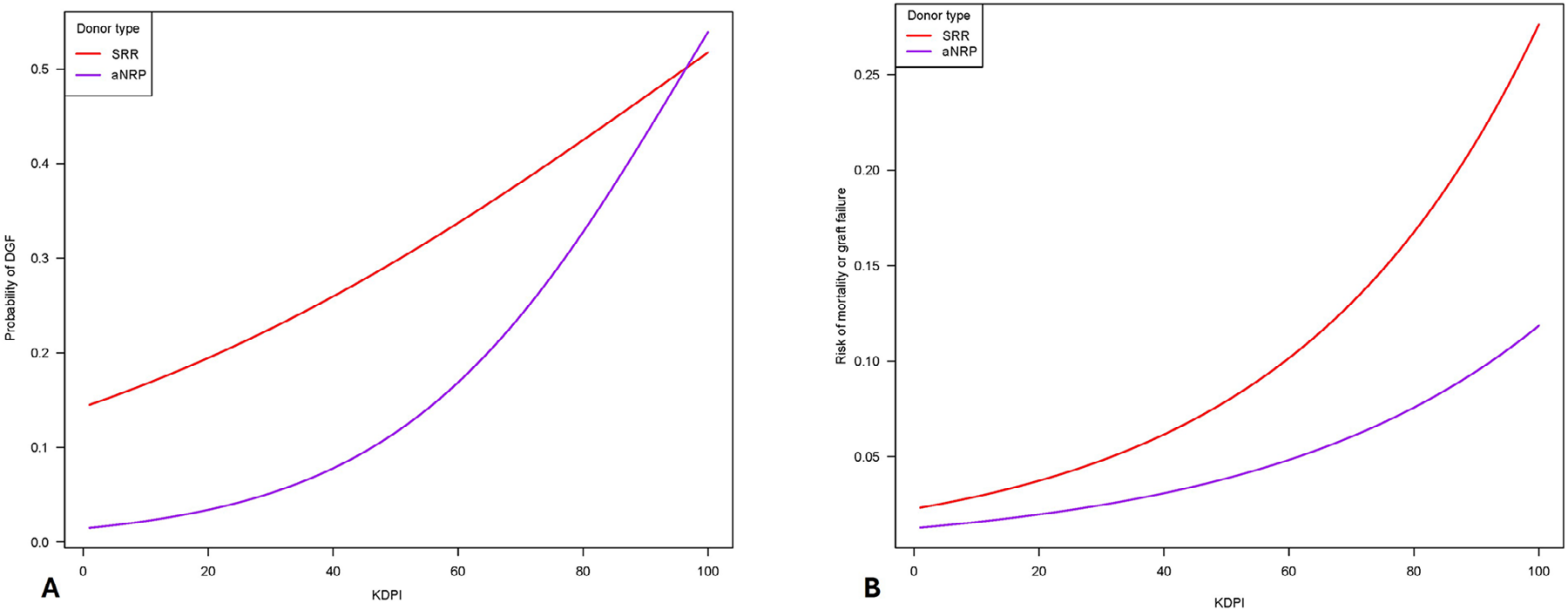
DCD‐restricted multivariable interaction models evaluating KDPI‐associated predicted outcomes by procurement strategy (SRR‐DCD vs aNRP‐DCD). (A). Predicted probability of delayed graft function by KDPI. (B). Predicted probability of 1‐year all‐cause graft loss by KDPI. aNRP‐DCD, abdominal normothermic regional perfusion donation after circulatory death; DCD, donation after circulatory death; DGF, delayed graft function; KDPI, kidney donor profile index; SRR‐DCD, super‐rapid recovery donation after circulatory death.

##### 3.5.5.2 KDPI Concordance With 1‐Year Overall Graft Survival in the DCD‐Restricted Model

KDPI did not show significant association with 1‐year overall graft survival in either group (HR 1.025 per 1% increase, 95% CI 0.987–1.065; *p* = 0.192). There was no statistical evidence that the KDPI/outcome relationship differed between SRR‐DCD and aNRP‐DCD kidneys (KDPI × donor type HR 0.997, 95% CI 0.928–1.072; *p* = 0.944). (Figure 2B).

## 4 Discussion

In this cohort of kidney transplants procured using a uniformly executed aNRP‐DCD technique, we found that procurement strategy may meaningfully modify early graft‐function risk in DCD kidneys; aNRP kidneys were associated with more favorable early graft function than SRR‐DCD despite higher KDPI. One‐year overall graft survival was similar across procurement groups, although interpretation is limited by the small number of events. In the DCD‐restricted analysis, KDPI remained positively associated with DGF overall, but the modeled KDPI–DGF relationship appeared more pronounced in SRR‐DCD than in aNRP‐DCD kidneys, although the formal interaction was not statistically significant. These findings are notable because this study isolates only abdominal NRP within a uniformly applied protocol. Taken together, the results suggest a favorable association between aNRP and early graft function while raising the possibility that current donor‐risk metrics may not fully capture procurement‐related differences in early clinical outcomes.

The observed reduction in DGF with aNRP relative to SRR is consistent with prior reports demonstrating attenuation of ischemia‐reperfusion injury with NRP in DCD procurement. In the Spanish national experience, SRR‐DCD was associated with nearly twice the odds of DGF compared with NRP‐DCD [29]. Similarly, registry‐based analyses in the United States have reported lower DGF rates among NRP‐DCD recipients compared with SRR‐DCD [9, 13, 14]. An Organ Procurement Organization (OPO)‐based cohort using a standardized NRP protocol also demonstrated lower DGF rates with NRP‐DCD compared to SRR‐DCD, with outcomes approaching those of BDD kidneys [10]. Consistent with these studies, short‐term patient and graft survival in our cohort were similar across procurement strategies showing stronger separation in early graft function than in short‐term survival outcomes [9, 12, 15]. The more favorable aNRP early graft function observed in the literature is biologically plausible, as NRP may partially reverse the metabolic and endothelial consequences of donor warm ischemia [8].

Interpretation of prior studies has been limited by heterogeneity in procurement techniques and inconsistent identification of NRP cases. Published U.S. registry‐based analyses relied on indirect inference to identify NRP donors because national datasets did not explicitly distinguish NRP from SRR‐DCD procurement until late 2025; even now, they do not reliably differentiate abdominal from thoracoabdominal NRP [9, 12, 13, 18]. These limitations introduce misclassification and make it difficult to isolate procurement‐specific effects. Also, registry data lack granular information on run‐level perfusion parameters, which may vary substantially across programs, and do not capture unsuccessful NRP runs, further limiting interpretation. By focusing on a homogeneous cohort of aNRP cases performed with a uniform technique with direct linkage to run‐level data, the present study reduces heterogeneity and allows clearer assessment of aNRP‐specific kidney outcomes.

We deliberately restricted this analysis to aNRP to avoid conflating physiologically distinct procurement strategies. TANRP donors are often selected for potential cardiac recovery and therefore represent a clinically different donor population, typically younger and lower risk than aNRP donors [13, 14]. TANRP restores pulsatile circulation through reestablishment of cardiac activity, whereas aNRP relies on extracorporeal, non‐pulsatile perfusion. This distinction may be clinically relevant, as non‐pulsatile flow has been associated with endothelial dysfunction and acute kidney injury in non‐pulsatile extracorporeal membrane oxygenation (ECMO) support settings [17]. Restricting the analysis to aNRP therefore provides a more consistent physiological framework for interpreting outcomes. Notably, this likely contributed to our exploratory analysis not identifying independent associations between individual aNRP run parameters and DGF or bilateral kidney nonuse.

KDPI score is calculated to estimate relative long‐term graft survival, not to directly predict DGF or short‐term graft function. However, OPTN also describes KDPI as summarizing “into a single number the quality of deceased donor kidneys relative to other recovered kidneys” [19]. KDPI replaced the “standard‐criteria” versus “expanded‐criteria” donor classification in 2014 and has since become a tool for clinical assessment of deceased‐donor kidney quality at the time of organ offer, thus influencing acceptance decisions. Practically, these decisions extend beyond estimating long‐term graft survival and include consideration of short‐term outcomes like DGF, which has a profound clinical and logistic impact that may extend beyond the early post‐transplant period. In the meta‐analysis by Li et al.,[6] DGF was associated with increased 1‐year graft failure and acute rejection. DGF also adds a substantial resource burden; Kim et al. reported that DGF was associated with longer hospitalization, higher costs of testing, monitoring and dialysis management in the immediate post‐transplant period [5].

Recent literature has renewed concern about KDPI interpretation in clinical use. One concern is the possible labeling effect, whereby kidneys labeled as high KDPI may be less likely to be accepted or utilized [20, 21, 30]. Therefore, although DGF should not be interpreted as a surrogate for KDPI's intended long‐term endpoint, the relationship between KDPI and early graft function remains clinically relevant, especially in DCD kidneys because KDPI incorporates DCD status as a static risk component without accounting for aNRP versus SRR procurement techniques, despite the more favorable early graft function reported with aNRP in prior studies [19]. In our DCD‐restricted multivariable models, aNRP was associated with lower odds of DGF after adjustment for KDPI, supporting the importance of evaluating recovery strategy alongside donor‐risk indices. In interaction analyses, higher KDPI remained associated with increased DGF risk overall, and modeled probability curves suggested lower DGF risk with aNRP compared with SRR at a given KDPI level, although the formal KDPI × donor‐type interaction did not reach statistical significance. This hypothesis‐generating pattern supports careful consideration of aNRP‐DCD kidney offers, including those with higher KDPI, rather than relying on KDPI alone when assessing early graft‐function risk.

For 1‐year overall graft survival, neither KDPI nor the KDPI × donor‐type interaction was significantly associated with outcomes. These findings suggest that procurement‐related differences may be most apparent in early graft function, whereas longer‐term outcomes may require larger cohorts and extended follow‐up to detect meaningful differences.

A major strength of this study is restricting the NRP cohort to abdominal‐only, using a uniformly executed protocol with direct linkage to run‐level data, thereby minimizing physiological heterogeneity and misclassification inherent in registry‐based analyses. This design also enabled focused evaluation of KDPI performance within a controlled procurement context. However, several limitations should be acknowledged. The local aNRP design and protocol standardization limit generalizability to other settings, and limit examining outcomes against variability in the aNRP protocols. The sample size is modest, and follow‐up is relatively short, limiting power to detect differences in longer‐term outcomes. In addition, because kidneys from this cohort were transplanted at multiple institutions, recipient outcomes were limited to STAR file reported fields. Accordingly, we could not evaluate more granular recipient outcome variables not captured in STAR file to examine the true burden of DGF in our cohort. The retrospective design precludes causal inference and leaves the possibility of residual confounding despite multivariable adjustment. Potential ex‐situ kidney normothermic machine perfusion (NMP) use at accepting centers or through third‐party platforms could not be reliably assessed from the available OPTN STAR data. NMP was not part of the local recovery protocol during the study period.

## 5 Conclusion

In this uniform aNRP kidney transplant cohort, aNRP‐DCD kidneys were associated with lower adjusted odds of DGF than SRR‐DCD despite higher donor age and baseline risk. Our findings suggest that at the identical KDPI values they are currently assigned, aNRP‐DCD kidneys may have a lower risk of DGF than SRR‐DCD kidneys. To our knowledge, this is the first study to evaluate this relationship in a homogeneous aNRP kidney cohort.

Standardization of aNRP implementation nationwide and incorporation of procurement technique into donor‐risk assessment may improve organ‐offer decision making by supporting more accurate risk assessment thus potentially reducing nonuse. Proper documentation of NRP run parameters in national registries will facilitate larger studies with longer follow‐up to determine whether the findings represent true procurement‐specific differences and whether early advantages observed with aNRP‐DCD kidneys translate into long‐term graft benefit.

## Author Contributions

Motaz A. Selim, MD, PhD: Participated in research design, data acquisition, data collection, data analysis, interpretation of results, and writing of the paper. Michelle Buff, MD: Participated in research design, interpretation of results, and critical review of the final manuscript. Michael Fenlon, MD: Participated in research design, interpretation of results, and critical review of the final manuscript. Austin Forbes, DO: Participated in data collection, interpretation of results, and critical review of the final manuscript. Chinedu Nwaduru, MBBCh: Participated in data collection, interpretation of results, and critical review of the final manuscript. Leonardo Aviles Ovalle: Participated in data collection, interpretation of results, and critical review of the final manuscript. Rebecca Kohler, PA‐C: Participated in interpretation of results and critical review of the final manuscript. Joshua J. Horns, PhD: Participated in research design, data analysis, interpretation of results, and critical review of the final manuscript. Jean Botha, MD: Participated in research design, interpretation of results, and critical review of the final manuscript. Michael A. Zimmerman, MD: Participated in research design, interpretation of results, writing of the paper, and critical review of the final manuscript.

## Funding

This research was supported by the Surgical Population Analysis Research Core (SPARC) in the Department of Surgery at the University of Utah. The computational resources used were partially funded by the NIH, Share Instrumentation Grant 1S10OD021644‐011A1.

## Disclosure

The authors have no disclosures.

## Conflicts of Interest

The authors declare no conflicts of interest.

## Artificial Intelligence (AI) Utilization Disclaimer

AI tools were used solely for language and writing refinement. AI did not contribute to study design, hypothesis generation, data analysis, interpretation of results, or production of tables or figures. All scientific content and conclusions were developed and verified by the authors. Authors are fully responsible for the content of the manuscript.

## Supporting information




**Supporting File 1**: ctr70729‐sup‐0001‐SuppMat.docx.

## Data Availability

The data that support the findings of this study are available on request from the corresponding author. The data are not publicly available due to privacy or ethical restrictions.
