# Supplementary material for: Early Transplant Outcomes and Kidney Donor Profile Index Risk Prediction Performance in Kidneys Recovered Using a Uniform Abdominal Normothermic Regional Perfusion Technique
Source: Clin Transplant. 2026 Oct 23;40(10):e70729. doi: 10.1111/ctr.70729 (PMC13693889; doi:10.1111/ctr.70729)
Supplement: Supplementary file 1 [file CTR-40-e70729-s001.docx]

**Supplemental Figure S1. Donor-level cohort construction and derivation of the final kidney transplant outcome cohort.**

The initial donor-level cohort included 400 donors recovered from a single donation service area, January 2023–December 2024. Donors with incomplete aNRP runs (converted to SRR) or aborted aNRP runs, TANRP recovery, en-bloc or dual kidney transplantation, kidneys not offered, or bilateral kidney nonuse were excluded. Completed aNRP donors in whom neither kidney was transplanted were excluded from transplant-outcome analyses but included in donor-level kidney nonuse analyses. Recipient data and transplant outcomes were obtained from deidentified OPTN STAR recipient outcome fields.

**Abbreviations:** aNRP, abdominal normothermic regional perfusion; BDD, brain-dead donor; DCD, donation after circulatory death; NRP, normothermic regional perfusion; DSA, donation service area; OPTN, Organ Procurement and Transplantation Network; SRR-DCD, super-rapid recovery donation after circulatory death; STAR, Standard Transplant Analysis and Research; TANRP, thoracoabdominal normothermic regional perfusion; UNOS, United Network for Organ Sharing.

**Donor Level: (n=400)**

Donors recovered from a single DSA, January 2023–December 2024

- Donor identified UNOS IDs were linked to local procurement records to identify aNRP donors and obtain granular NRP run parameters.
- Remaining donors and transplant data were obtained from deidentified OPTN STAR files.

Exclusions (n= 117)

- Incomplete aNRP run (n=3)
- TANRP (n=14)
- En-bloc or dual kidney transplants donors (n=13)
- Kidneys not offered (n=18)
- Donors with bilateral kidney nonuse (n=69)

Final utilized donor cohort:

Donors yielding at least one kidney transplant

(n= 283 donors)

BDD

- Donors (n=163)

- Kidney Transplants (n=310)

aNRP-DCD

- Donors (n=51)
- Kidney Transplants (n=91)

**Final Eligible Kidney Transplants (n=523)**

(Deidentified recipient/transplant data from STAR file)

SRR-DCD

- Donors (n=69)
- Kidney transplants (n=122)

**Supplemental Table S1. Kidney nonuse by procurement group**

Across the three study groups, the overall kidney nonuse rate was 181 out of 704 recovered kidneys (25.7%). Kidney nonuse differed significantly across procurement groups overall (p<0.001). Pairwise comparisons were performed using Fisher’s exact test and showed significantly lower kidney nonuse in BDD donors compared with SRR-DCD and aNRP-DCD donors, while kidney nonuse did not differ significantly between SRR-DCD and aNRP-DCD donors.

**Abbreviations:** aNRP-DCD, abdominal normothermic regional perfusion donation after circulatory death; BDD, brain-dead donor; SRR-DCD, super-rapid recovery donation after circulatory death.

|  | **Nonuse comparison,**  n nonuse/n total procured (%) | **p-value** |
| --- | --- | --- |
| BDD vs SRR-DCD | 66/376 (17.6%) vs 64/186 (34.4%) | <0.001 |
| BDD vs aNRP-DCD | 66/376 (17.6%) vs 51/142 (35.9%) | <0.001 |
| SRR-DCD vs aNRP-DCD | 64/186 (34.4%) vs 51/142 (35.9%) | 0.816 |

**Supplemental Table S2. Rejection episode within 6 and 12 months by procurement group.** Percentages for rejection episodes were calculated among recipients with nonmissing STAR file rejection data. Pairwise comparisons were performed using Fisher’s exact test.

|  | **aNRP-DCD** | **SRR-DCD** | **BDD** | **aNRP vs SRR p-value** | **aNRP vs BDD p-value** | **SRR vs BDD p-value** |
| --- | --- | --- | --- | --- | --- | --- |
| Treated rejection within 6 months, n (%) | 2/74 (2.7%) | 4/101 (4.0%) | 13/269 (4.8%) | 1.0 | 0.54 | 1.0 |
| Missing values (6 months), n (%) | 17/91 (18.7%) | 21/122 (17.2%) | 41/310 (13.2%) |  |  |  |
|  |  |  |  |  |  |  |
| Treated rejection within 12 months, n (%) | 3/53 (5.7%) | 5/88 (5.7%) | 19/218 (8.7%) | 1.0 | 0.58 | 0.48 |
| Missing values (12 months), n (%) | 38/91 (41.8%) | 34/122 (27.9%) | 92/310 (29.7%) |  |  |  |

**Supplemental Table S3. Exploratory KDPI-adjusted associations between aNRP run parameters and DGF or bilateral kidney nonuse.**

Models were fitted separately for each aNRP run parameter and adjusted for KDPI. Results are presented as odds ratios with 95% confidence intervals and p-values. These exploratory analyses were limited by the small number of completed aNRP donors and small number of events of DGF, thus should be interpreted cautiously.
**Abbreviations:** aNRP, abdominal normothermic regional perfusion; CI, confidence interval; DGF, delayed graft function; KDPI, Kidney Donor Profile Index.

A. Association between aNRP run parameters and DGF

| **aNRP run parameter** | **Odds ratio (95% CI)** | **p-value** |
| --- | --- | --- |
| Flow time, per 1-minute increase | 1.012 (0.992–1.032) | 0.250 |
| Average flow, per 1 L/min/m^2^ increase | 1.002 (0.439–2.287) | 0.996 |
| Starting lactate, per 1-unit increase | 1.083 (0.923–1.271) | 0.326 |
| Terminal lactate, per 1-unit increase | 1.030 (0.885–1.198) | 0.705 |
| Starting pH, per 1-unit increase | 0.137 (0.004–4.473) | 0.264 |
| Terminal pH, per 1-unit increase | 0.237 (0.005–11.062) | 0.463 |

B. Association between aNRP run parameters and bilateral kidney nonuse

| **aNRP run parameter** | **Odds ratio (95% CI)** | **p-value** |
| --- | --- | --- |
| Flow time, per 1-minute increase | 1.009 (0.986–1.032) | 0.447 |
| Average flow, per 1 L/min/m^2^ increase | 0.290 (0.076–1.103) | 0.069 |
| Starting lactate, per 1-unit increase | 1.116 (0.889–1.401) | 0.346 |
| Terminal lactate, per 1-unit increase | 1.144 (0.958–1.364) | 0.136 |
| Starting pH, per 1-unit increase | 0.127 (0.003–4.638) | 0.261 |
| Terminal pH, per 1-unit increase | 0.007 (0.000–0.932) | 0.047 |

**Supplemental Table S4**. **Exploratory KDPI sensitivity models for DGF in the DCD-restricted cohort.**

Models were restricted to DCD kidney transplant recipients, with SRR-DCD as the reference group. Effects are shown as odds ratios with 95% confidence intervals and p-values.

**Abbreviations:** aNRP, abdominal normothermic regional perfusion; DCD, donation after circulatory death; DGF, delayed graft function; KDPI, Kidney Donor Profile Index; SRR-DCD, super-rapid recovery donation after circulatory death.

A. KDPI continuous interaction model outputs for likelihood of **DGF.** Effect of donor type relative to SRR; effect of KDPI represents increase of 1.

| **Variable** | **Odds Ratio (95% CI)** | **p-value** |
| --- | --- | --- |
| Donor type (aNRP) | 0.088 (0.011-0.693) | **0.021** |
| KDPI (1 point increase) | 1.019 (1.002-1.036) | **0.025** |
| Donor type (aNRP) * KDPI | 1.025 (0.996-1.056) | 0.094 |

B. KDPI ≥85% threshold model outputs for likelihood of **DGF.** Effect of donor type relative to SRR; effect of KDPI ≥85%

| **Variable** | **Odds Ratio (95% CI)** | **p-value** |
| --- | --- | --- |
| Donor type (aNRP) | 0.495 (0.238-1.031) | 0.06 |
| KDPI ≥85 | 3.886 (1.232-12.252) | **0.021** |
| Donor type (aNRP) * KDPI 85+ | 1.01 (0.223-4.571) | 0.99 |

C. KDPI continuous interaction model outputs for likelihood of **DGF**. Effect of donor type relative to SRR; effect of KDPI represents increase of 20 points.

| **Variable** | **Odds Ratio (95% CI)** | **p-value** |
| --- | --- | --- |
| Donor type (aNRP) | 0.103 (0.013-0.813) | **0.031** |
| KDPI (20-point increase) | 1.41 (1.031-1.929) | **0.032** |
| Donor type (aNRP) * KDPI 20-point increase | 1.619 (0.906-2.893) | 0.104 |
